# Dual-Head Pix2Pix Network for Material Decomposition of Conventional CT Projections with Photon-Counting Guidance

**DOI:** 10.3390/s25195960

**Published:** 2025-09-25

**Authors:** Yanyun Liu, Zhiqiang Li, Yang Wang, Ruitao Chen, Dinghong Duan, Xiaoyi Liu, Xiangyu Liu, Yu Shi, Songlin Li, Shouping Zhu

**Affiliations:** 1 School of Life Science and Technology, Xidian University & Engineering Research Center of Molecular and Neuro Imaging, Ministry of Education, Xi’an 710026, China; yanyunliu@stu.xidian.edu.cn (Y.L.);; 2 Xi’an Key Laboratory of Intelligent Sensing and Regulation of Trans-Scale Life Information, School of Life Science and Technology, Xidian University, Xi’an 710071, China; 3 International Joint Research Center for Advanced Medical Imaging and Intelligent Diagnosis and Treatment, School of Life Science and Technology, Xidian University, Xi’an 710071, China; 4 Innovation Center for Advanced Medical Imaging and Intelligent Medicine, Guangzhou Institute of Technology, Xidian University, Guangzhou 510555, China; 5 School of Electrical Engineering and Automation, Suzhou University of Technology, Suzhou 215500, China; yushi@szut.edu.cn; 6 College of Electrical Engineering, Henan University of Technology, Zhengzhou 450001, China

**Keywords:** material decomposition, dual-head Pix2Pix, photon-counting detector, conventional energy-integrating detector, X-ray projection imaging, deep learning

## Abstract

Material decomposition in X-ray imaging is essential for enhancing tissue differentiation and reducing the radiation dose, but the clinical adoption of photon-counting detectors (PCDs) is limited by their high cost and technical complexity. To address this, we propose Dual-head Pix2Pix, a PCD-guided deep learning framework that enables simultaneous iodine and bone decomposition from single-energy X-ray projections acquired with conventional energy-integrating detectors. The model was trained and tested on 1440 groups of energy-integrating detector (EID) projections with their corresponding iodine/bone decomposition images. Experimental results demonstrate that the Dual-head Pix2Pix outperforms baseline models. For iodine decomposition, it achieved a mean absolute error (MAE) of 5.30 ± 1.81, representing an ~10% improvement over Pix2Pix (5.92) and a substantial advantage over CycleGAN (10.39). For bone decomposition, the MAE was reduced to 9.55 ± 2.49, an ~6% improvement over Pix2Pix (10.18). Moreover, Dual-head Pix2Pix consistently achieved the highest MS-SSIM, PSNR, and Pearson correlation coefficients across all benchmarks. In addition, we performed a cross-domain validation using projection images acquired from a conventional EID-CT system. The results show that the model successfully achieved the effective separation of iodine and bone in this new domain, demonstrating a strong generalization capability beyond the training distribution. In summary, Dual-head Pix2Pix provides a cost-effective, scalable, and hardware-friendly solution for accurate dual-material decomposition, paving the way for the broader clinical and industrial adoption of material-specific imaging without requiring PCDs.

## 1. Introduction

In computed tomography (CT), material decomposition has gained increasing attention beyond conventional morphological evaluation [1,2,3,4], as it enables the differentiation and quantification of specific substances such as iodine and calcium [5]. Iodine–bone separation plays an important role in vascular enhancement [6], bone mineral density assessment [7], and pathological calcification detection, thereby improving diagnostic accuracy and clinical decision-making [8,9,10]. Beyond medical applications, this technique has also been widely applied in industrial contexts. For example, dual-energy methods have been used to detect semi-precious beryl in surrounding rock, and multi-energy material separation combined with neural networks such as the YOLO algorithm has been employed to segment particles of different rock types [11,12]. The development of CT detectors has progressed from energy-integrating detectors (EIDs) to dual-energy CT (DECT) and more recently to photon-counting detectors (PCDs) [13]. EIDs lose energy information during acquisition, which limits their capability for material decomposition [14]. DECT alleviates this limitation to some extent but still suffers from an increased radiation dose, susceptibility to artifacts, and limited quantitative accuracy [15]. In contrast, PCDs directly detect individual X-ray photons while preserving their spectral information, offering significant advantages in energy resolution, noise suppression, and multi-material decomposition [9,16,17,18]. These benefits have been demonstrated in preclinical and early clinical studies. However, the high cost and technical complexity of PCDs have restricted their large-scale clinical adoption [19], leaving EIDs as the mainstream in current practice. This situation highlights the importance of improving the material decomposition performance on EID systems [1].

With the rapid development of deep learning, especially generative adversarial networks [20,21,22,23], new opportunities have emerged for learning the complex mapping between projection or image data and decomposed material maps. Most existing studies focus on image-domain decomposition [1,24,25]. However, such methods are highly dependent on spectral fidelity, are prone to artifacts, and often lack quantitative accuracy. By contrast, projection-domain data preserve richer structural and spectral information. Direct modeling in the projection domain introduces physical constraints at an early stage, which can reduce error accumulation, suppress beam-hardening effects, and compensate for detector- and acquisition-related distortions.

Projection-domain decomposition in PCD-CT is typically performed by representing measured projection data as linear combinations of basic materials (e.g., soft tissue, bone, and iodine) [26], followed by independent reconstruction. Compared with image-domain approaches, this strategy provides a stronger physical consistency [27]. Prior studies have proposed regularization and filtering strategies to mitigate noise propagation [28], as well as multi-stage or hierarchical optimization frameworks to enhance quantitative accuracy and robustness against artifacts [29]. More recently, machine learning-based approaches have been introduced to fully exploit the high-dimensional features embedded in projection data while retaining physical constraints [30]. Experimental results indicate that projection-domain methods can explicitly compensate for scattering, noise, and detector response while improving reconstruction and segmentation quality [27]. Furthermore, projection-domain decomposition has shown superior performance in both non-K-edge and K-edge material quantification, suggesting the broad potential for clinical applications. Leveraging the high-quality decomposition results obtained from PCD provides a promising pathway to enhance material decomposition in EID systems, which serves as the central motivation of this study.

In this study, we will first establish a PCD-CT-based data acquisition platform to construct a dedicated iodine–bone separation projection dataset. Building upon this, we will design an enhanced Pix2Pix-based generative model with a dual-head output architecture to enable accurate mapping from single-channel projections to dual-material decomposition. The effectiveness of the proposed approach will be further evaluated using projection data from conventional EID systems.

## 2. System Setup and Data Acquisition

### 2.1. System Architecture

**Figure 1:**
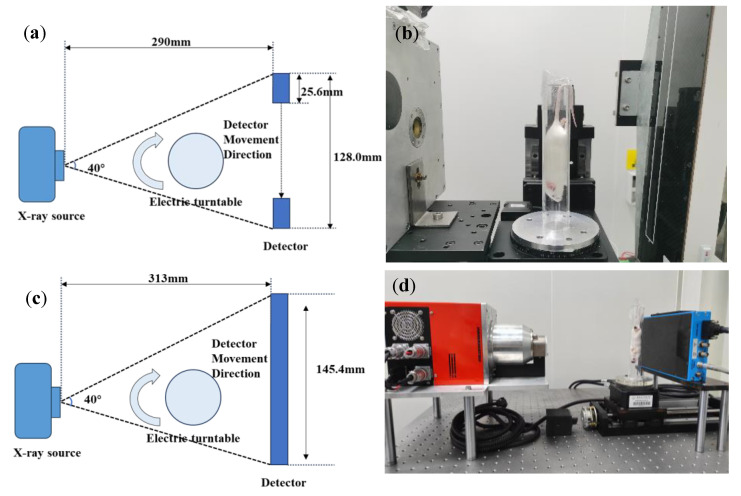
Overview of the PCD-CT and EID-CT imaging system, (**a**) is the schematic of the PCD-CT system geometry and (**b**) is the photograph of the PCD-CT physical system, and (**c**) is the schematic of the EID-CT system geometry and (**d**) is the photograph of the EID-CT physical system.

To enable both supervised training and cross-domain evaluation, we constructed a dual-energy projection acquisition system based on a PCD-CT platform and additionally acquired data using a conventional micro-CT system equipped with an EID. The PCD-CT system is illustrated in Figure 1.

Figure 1a presents a schematic diagram of our PCD-CT system, highlighting key geometric parameters such as the source-to-detector distance and imaging field of view. Figure 1b shows a photograph of the actual system setup. This PCD-CT system integrates an RZX-8016D X-ray source (maximum 80 kV, 1000 μA) and an XCounter FX3 PCT (CdTe-CMOS sensor, XCounter AB, Sweden). The FX3 detector features a dual-energy CdTe photon-counting sensor with a pixel size of 100 μm, enabling energy-resolved data acquisition. To acquire multi-angle projection data, the system operates in TDS (Translation During Scan) mode, in which the detector is translated horizontally using a high-precision PSA150-11-X motorized stage, allowing for high-resolution projection imaging at multiple viewing angles. For comparison, a conventional micro-CT system (Figure 1c,d) was used to acquire projection data using a Dexela 1512 flat-panel detector (Varex Imaging) with a pixel size of 74.8 μm. This EID-based system performs energy integration over the entire X-ray spectrum and does not support spectral resolution. To maintain spectral consistency across modalities, the same X-ray source was integrated into both the PCD-based and EID-based imaging systems.

### 2.2. Image Acquisition and Preprocessing

All animal experiments were conducted in strict accordance with the ethical regulations and approval procedures of the Animal Ethics Committee of Xi’an Medical University. Four female BALB/c nude mice (6–8 weeks old, 18–20 g) were used for in vivo imaging. Prior to scanning, each mouse was anesthetized via an intraperitoneal injection of 100 μL chloral hydrate. Then, 150 μL of iohexol contrast agent (150 mg/mL) was administered via the tail vein. Following injection, the mice were immobilized in a custom-designed acrylic cylindrical holder to minimize motion artifacts during imaging. Each mouse underwent a full 360° scan with 1° angular increments, resulting in 360 projection images per subject. In total, 1440 projection images were collected from four mice. Based on predefined energy thresholds, the acquired data were processed as follows:

The PCD records the energy of each detected photon, and according to the XCounter FX3 instructions, we set energy thresholds to obtain projections at different energy levels. Projections acquired at a 10 keV threshold were used as conventional projections (ConvProj). Projections at a 30 keV threshold served as high-energy projections. Material decomposition was performed using the energy silhouette subtraction method [31], where subtraction between energy channels yielded bone-preserving projections (BoneProj) and iodine-preserving projections (IodineProj).

All projection images were saved in a standard 8-bit grayscale PNG format, with a resolution of 1024 × 512 pixels and a pixel size of 100 μm. For each subject, 80% of the images were used for model training and 20% for testing. To further evaluate the cross-domain generalization capability of the proposed method, additional projection data were collected using the conventional energy-integrating micro-CT system described in Section 2.1. The original images (1536 × 1944 pixels, pixel size 74.8 μm) were preprocessed and center cropped to 1024 × 512 pixels, matching the resolution of the training dataset. These external data were used to assess the material decomposition performance of the trained model on a different imaging domain.

## 3. Methodology

**Figure 2:**
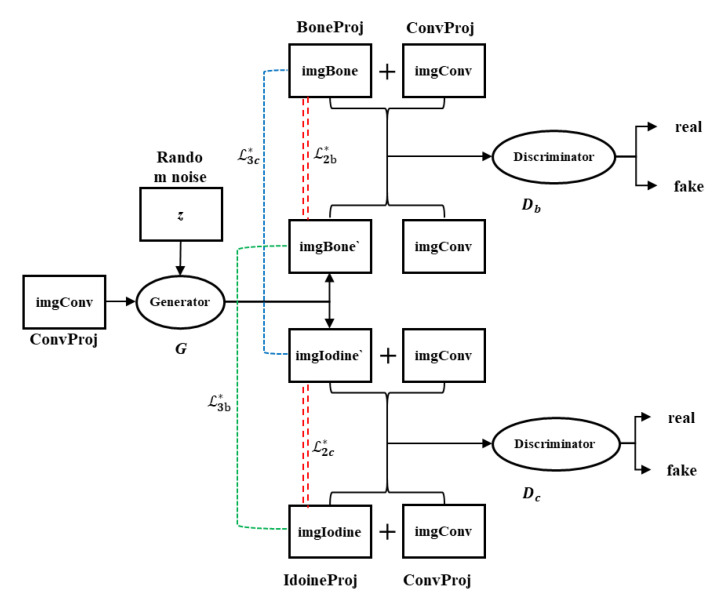
Overview of the Dual-branch Pix2Pix network.

As shown in Figure 2, the input imgConv from ConvProj, along with a random noise vector *z*, is fed into the generator *G*. The generator simultaneously produces two output images, imgBone ‘and imgIodine’, corresponding to the bone domain (BoneProj) and the iodine domain (IdoineProj), respectively. These generated images are then concatenated with the original input imgConv, and the real imgBone and imgIodine are fed into the corresponding discriminators, *D*_*b*_ and *D*_*c*_, for real/fake classification. The corresponding loss components are indicated with dashed lines in Figure 2 and are described in detail in Section 3.3. In the following, we provide a detailed description of each component of the proposed network.

### 3.1. Dual-Head Pix2Pix Generative Network Architecture

**Figure 3:**
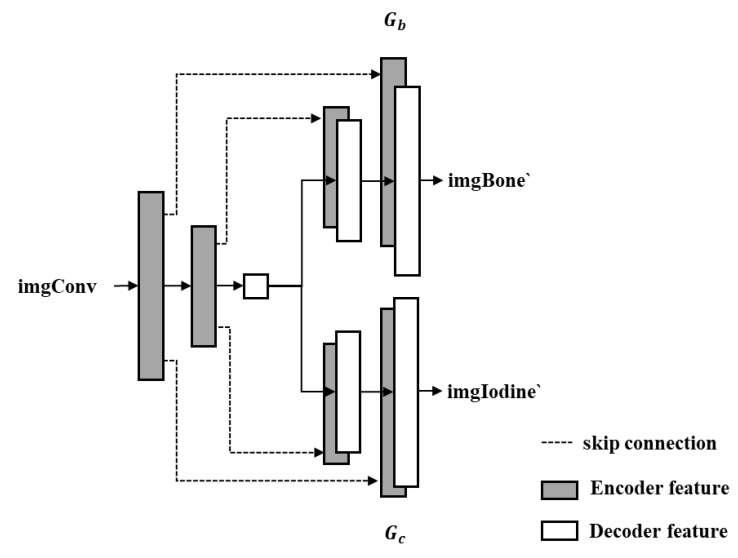
Dual-head image generator.

This study proposes an enhanced generator architecture based on U-Net, termed Dual-head Pix2Pix, which adopts an encoder–decoder framework (Figure 3). The encoder maps the input image into a low-dimensional latent representation, while the decoder reconstructs the target output via transposed convolutions. Compared with the conventional U-Net, the proposed architecture introduces dual-decoder branches, each responsible for independently generating images corresponding to BoneProj (bone structures) and IodineProj (iodine contrast agent), thereby enabling the simultaneous prediction of multiple material components.

As illustrated in Figure 3, the input comprises the imgConv and random noise *z*. These inputs are first passed through a shared encoder to extract latent features, which are then fed into two parallel decoders. Decoder *G*_*b*_ is responsible for generating the output imgBone ‘corresponding to BoneProj, while decoder *Gc* generates the output imgIodine’ for IodineProj. To retain high-frequency spatial details and improve the reconstruction accuracy, skip connections (depicted as dashed lines in Figure 3) are implemented between corresponding layers of the encoder and each decoder.

This dual-decoder architecture enables the generator to effectively learn a mapping from a single input image, conditioned on a specific label, to two distinct image domains, making it particularly well-suited for material decomposition tasks in CT projection imaging.

### 3.2. Dual Discriminator Design Based on PatchGAN

The discriminator in the proposed Dual-head Pix2Pix network adopts the PatchGAN architecture from the original Pix2Pix framework [32], which divides the input image into multiple local patches and performs a real/fake classification on each patch independently. The key difference in our design is the introduction of two separate discriminators, $D_{b}$ and $D_{c}$ (Figure 2), which independently evaluate the generator’s outputs corresponding to BoneProj and IodineProj, respectively. Compared with a global image-level discriminator, PatchGAN uses significantly fewer parameters. This improves the training stability and convergence, especially for high-resolution images. Its low computational complexity allows for the use of multiple discriminators without substantially increasing the overall computational burden. This effectively reduces both the training cost and inference time. As illustrated in Figure 2, both the generated image and the Ground Truth are concatenated with the original input image (imgConv) along the channel dimension before being fed into the discriminator. This design implements conditional discrimination, aiming to enable the discriminator to evaluate whether the generated image is both “realistic” and “consistent” with the conditional input rather than merely assessing the authenticity of a single image. Consequently, this approach ensures that the generator produces outputs that are not only visually plausible but also semantically aligned with the input condition.

### 3.3. Loss Function Design

The loss function in the Dual-head Pix2Pix network comprises three components (Figure 2): (1) an adversarial loss $L_{1}^{*}$ to ensure realism of the generated outputs, (2) a reconstruction loss $L_{2}^{*}$ to enforce pixel-wise similarity with the Ground Truth, and (3) a mutual exclusivity loss $L_{3}^{*}$ to exploit the complementary nature of iodine and bone decomposition images.

Adversarial Loss $L_{1}^{*}$

Dual-head Pix2Pix introduces two adversarial branches by modifying the output channels. The adversarial loss for BoneProj is formulated as follows:

(1) $$L_{1b}^{*}=E_{a,b}\left[\log D_{b}\left(a,b\right)\right]+E_{a,z}\left[\log\left(1-D_{b}\left(a,G_{b}\left(a,z\right)\right)\right)\right]$$

Similarly, for IodineProj,

(2) $$L_{1c}^{*}=E_{a,c}\left[\log D_{c}\left(a,c\right)\right]+E_{a,z}\left[\log\left(1-D_{c}\left(a,G_{c}\left(a,z\right)\right)\right)\right]$$

The total adversarial loss is the sum of the two branches:

(3) $$L_{1}^{*}=\arg{min}_{G_{b}}{max}_{D_{b}}L_{1b}^{*}+\arg{min}_{G_{c}}{max}_{D_{c}}L_{1c}^{*}$$

Reconstruction Loss $L_{2}^{*}$

To ensure the generated images closely resemble the Ground Truth, an $L_{1}$ loss is employed. For BoneProj,

(4) $$L_{2b}^{*}=E_{a,b,z}\left[\log\left\|b-G_{b}\left(a,z\right)\right\|\right]$$

And for IodineProj,

(5) $$L_{2c}^{*}=E_{a,c,z}\left[\log\left\|c-G_{c}\left(a,z\right)\right\|\right]$$

The total reconstruction loss is

(6) $$L_{2}^{*}=L_{2b}^{*}+L_{2c}^{*}$$

Mutual Exclusivity Loss $L_{3}^{*}$

To enforce the separation of features between iodine and bone components, a mutual exclusivity loss is introduced. First, the inverse of the $L_{1}$ difference is used as a base metric:

(7) $$L_{3b}^{*}=E_{a,b,z}\left[\log\left\|c-G_{b}\left(a,z\right)\right\|\right]$$

(8) $$L_{3c}^{*}=E_{a,c,z}\left[\log\left\|b-G_{c}\left(x,z\right)\right\|\right]$$

To ensure positivity and stability, a Sigmoid function is applied:

(9) $$L_{3}^{*}=1-Sigmoid\left(L_{3b}^{*}\right)+1-Sigmoid\left(L_{3c}^{*}\right)$$

When the predicted outputs for the mutually exclusive iodine and bone channels are well-separated, this loss approaches zero, encouraging disentangled material decomposition.

Objective Function

The overall objective function of Dual-head Pix2Pix is defined as follows:

(10) $$L^{*}=\lambda_{1}L_{1}^{*}+\lambda_{2}L_{2}^{*}+\lambda_{3}L_{3}^{*}$$

where $\lambda_{i}(i=1,2,3)$ are the weighting coefficients for each loss component. In our experiments, we set $\lambda_{1}=1$, $\lambda_{2}$ = 100, and $\lambda_{3}=100$. The mutual exclusivity loss $L_{3}^{*}$ acts as a reward term that guides the generator to improve the contrast between the decomposition outputs, enhancing the overall material separation performance.

### 3.4. Training Strategy and Parameter Settings

All experiments were conducted on a computing platform running Ubuntu 22.04, equipped with an AMD Ryzen 5950X CPU, 128 GB of RAM, and an NVIDIA GeForce RTX 4090 GPU. The neural network used in this study was implemented using the PyTorch 1.5+ framework, with structural modifications carried out via the MMEditing toolbox from the open-source OpenMMLab project. Both the generator and the discriminators were optimized using the Adam optimizer. During training, each batch was constructed by randomly sampling data from both the source and target domains. The batch size was set to 4, and the training was conducted for a total of 333 epochs.

To improve training stability and accelerate convergence, a warm-up learning rate strategy was employed. Specifically, the learning rate was initialized at 0 and linearly increased over the first 10,000 iterations to a peak value of 9 × 10^−4^. After the warm-up phase, a linear decay strategy with a decay factor of 0.9 was applied, and the minimum learning rate was constrained to 5 × 10^−6^ to maintain stability throughout training.

### 3.5. Baselines and Metrics

Both qualitative and quantitative evaluations were performed to assess the effectiveness of the proposed method. To further validate its generalization capability, we conducted comparative experiments against multiple generative models and additionally evaluated the performance on external projection images acquired from an EID-CT system. To comprehensively assess the quality of material decomposition images generated by Dual-head Pix2Pix, the following quantitative metrics were used:

Mean Absolute Error (MAE)

The MAE measures the average absolute difference between the generated image and the Ground Truth:

(11) $$MAE=\frac{1}{n}\sum_{i=1}^{n}\left|{\hat{y}}_{i}-y_{i}\right|$$

Multi-Scale Structural Similarity Index (MS-SSIM)

MS-SSIM evaluates the structural similarity over multiple image scales, defined as follows:

(12) $$MS-SSIM={\left[l\left(\hat{y},y\right)\right]}^{\alpha_{M}}\cdot \prod_{j=1}^{M}{\left[c\left(\hat{y},y\right)\right]}^{\beta_{j}}\cdot {\left[s\left(\hat{y},y\right)\right]}^{\gamma_{j}}$$

Pearson Correlation Coefficient (Pearson-R)

This metric evaluates the linear correlation between the predicted and reference images:

(13) $$r=\frac{\sum_{i=1}^{n}\left({\hat{y}}_{i}-\hat{Y}\right)\left(y_{i}-Y\right)}{\sqrt{\sum_{i=1}^{n}{\left({\hat{y}}_{i}-\hat{Y}\right)}^{2}}\sqrt{\sum_{i=1}^{n}{\left(y_{i}-Y\right)}^{2}}}$$

Peak Signal-to-Noise Ratio (PSNR)

PSNR reflects the similarity between two images based on the mean squared error (MSE):

(14) $$MSE=\frac{1}{mn}\sum_{i=0}^{m-1}\sum_{j=0}^{n-1}{\left[y\left(i,j\right)-\hat{y}\left(i,j\right)\right]}^{2}$$

Given the maximum pixel value ${MAX}_{y}$, PSNR is defined as follows:

(15) $$PSNR=10\cdot \log_{10}\frac{{\left({MAX}_{y}\right)}^{2}}{MSE}$$

Statistical analysis was performed using paired *t*-tests to compare the proposed method with the baseline models. Results were considered statistically significant at *p* < 0.05 and extremely significant at *p* < 0.0001. To further evaluate the material-specific performance, we extracted line profiles across selected regions of interest (ROI). Sampling lines were drawn on the raw image a in areas known to contain only bone or only iodine. Corresponding lines were then evaluated on the bone image b and iodine image ccc to analyze the material separation quality and intensity preservation.

In addition, to verify the accuracy of iodine–bone separation, regions of interest were selected along the line profiles in the iodine and bone domains, and the pixel intensity distribution curves were plotted along the specified paths.

## 4. Results

To comprehensively evaluate the proposed method, the Results section first presents comparisons with different models to assess the relative performance, followed by cross-domain testing on traditional EID projection images to examine the model’s generalization capability. Finally, an ablation study on the loss functions in the Dual-head Pix2Pix network is conducted.

### 4.1. Comparison with Different Models

To comprehensively validate the superiority of the proposed method, we conducted comparative experiments using the same training and testing datasets on CycleGAN, Pix2Pix, and the proposed Dual-head Pix2Pix network.

Table 1 presents quantitative results for iodine decomposition using different methods. Compared to the original input images, the proposed Dual-head Pix2Pix model reduces the MAE from 51.07 to 5.30 (a reduction of 89.6%), improves the MS-SSIM from 0.80 to 0.91, increases the Pearson correlation coefficient from 0.91 to 0.99, and raises the PSNR from 13.01 dB to 32.06 dB. Compared with CycleGAN and standard Pix2Pix, Dual-head Pix2Pix consistently outperforms across all metrics, demonstrating its superior ability to preserve iodine details while suppressing bone interference.

Table 2 shows quantitative results for bone decomposition. Relative to the input images, Dual-head Pix2Pix reduces the MAE by 58.1% (from 22.77 to 9.55), improves the MS-SSIM from 0.78 to 0.84, maintains the Pearson-R at 0.98, and increases the PSNR from 19.72 dB to 26.74 dB. Dual-head Pix2Pix also surpasses CycleGAN and standard Pix2Pix in all metrics, highlighting its improved capacity for accurate bone structure reconstruction and iodine artifact suppression.

**Figure 4:**
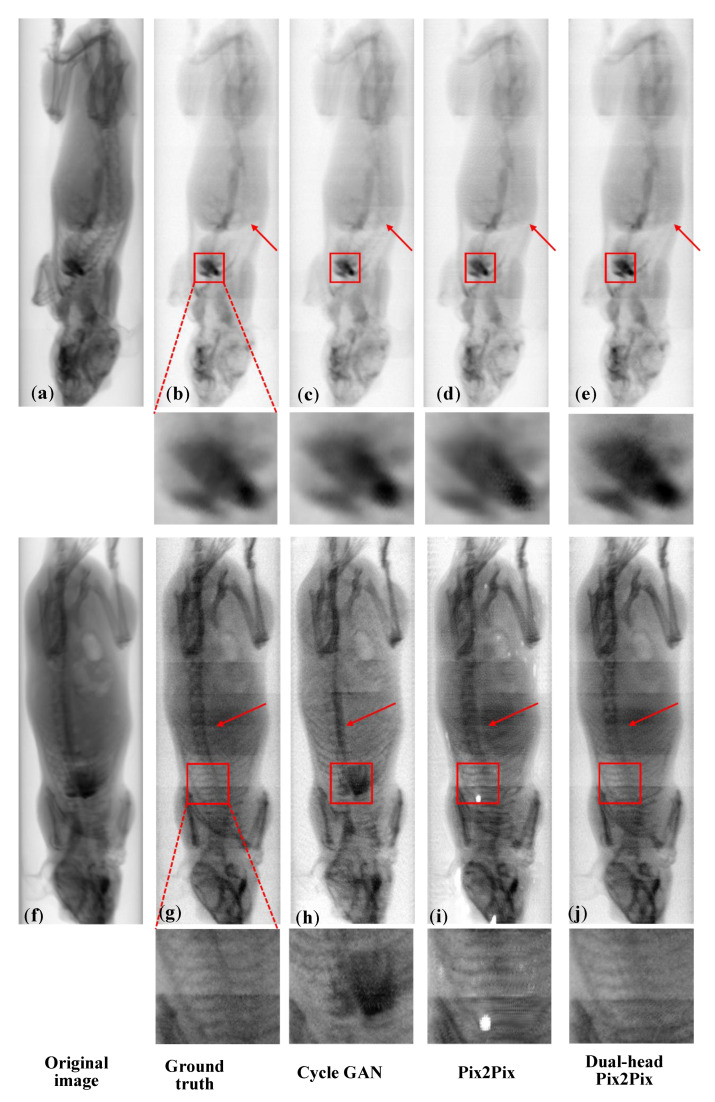
Material decomposition results obtained with different algorithms. The first row (**a**–**e**) shows iodine decomposition, and the second row (**f**–**j**) shows bone decomposition. From left to right: (**a**,**f**) original images, (**b**,**g**) Ground Truth, (**c**,**h**) CycleGAN results, (**d**,**i**) Pix2Pix results, and (**e**,**j**) Dual-head Pix2Pix results. Red arrows indicate bone-enriched regions, and red boxes highlight iodine-enriched regions.

As shown in Figure 4, for iodine decomposition, red arrows indicate residual bone structures. CycleGAN outputs retain visible spine components, whereas Pix2Pix and Dual-head Pix2Pix effectively suppress bone interference. For iodine detail preservation, the enlarged cardiac region (red box) shows that Dual-head Pix2Pix produces more natural textures and is visually closer to the Ground Truth than Pix2Pix, demonstrating the benefit of the dual-decoder design for fine-grained iodine reconstruction. For bone decomposition, red boxes mark regions with a high iodine concentration. Pix2Pix struggles to recover fine bone structures, often exhibiting unnatural textures or small “white spot” artifacts. In contrast, Dual-head Pix2Pix achieves more accurate structural recovery, preserves bone integrity more faithfully, and generates more natural textures, even under strong iodine interference.

**Figure 5:**
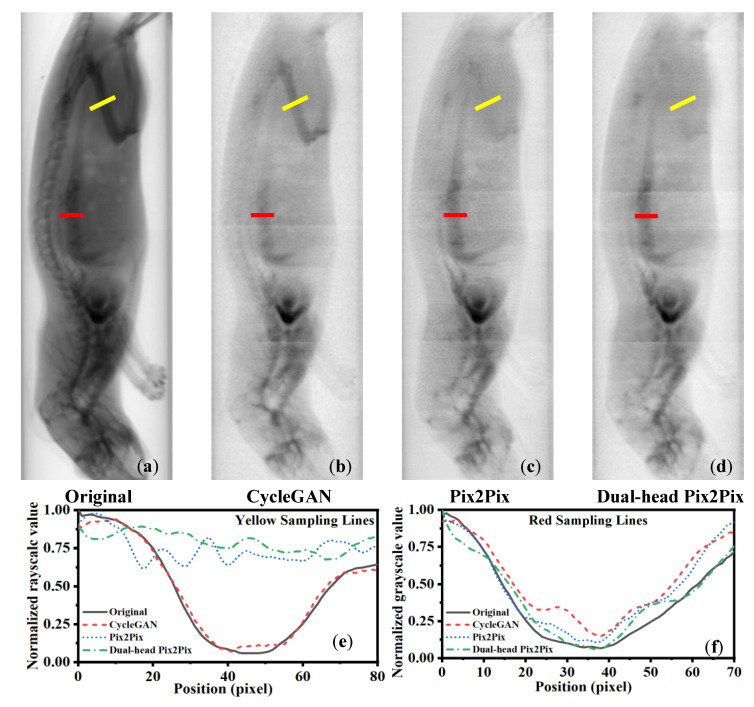
Line profile analysis of ROIs in iodine decomposition results obtained with different algorithms. (**a**) Original input; (**b**) result from CycleGAN; (**c**) result from Pix2Pix; and (**d**) result from Dual-head Pix2Pix. The yellow and red lines in (**a**–**d**) indicate the selected ROIs, where the yellow line corresponds to the bone region and the red line corresponds to the iodine region. (**e**) Line profile of the yellow ROI; (**f**) line profile of the red ROI.

Figure 5 shows the region-specific line profile analysis for iodine decomposition using different algorithms. The yellow and red sampling lines mark bone-rich and iodine-rich regions, respectively. In the yellow line profiles (e), greater deviation from the original input indicates better bone suppression; the Dual-head Pix2Pix output shows the largest deviation, confirming superior suppression. In the red line profiles (f), smaller deviation reflects better iodine preservation. The Dual-head Pix2Pix aligns most closely with the input, demonstrating the best retention of iodine details.

**Figure 6:**
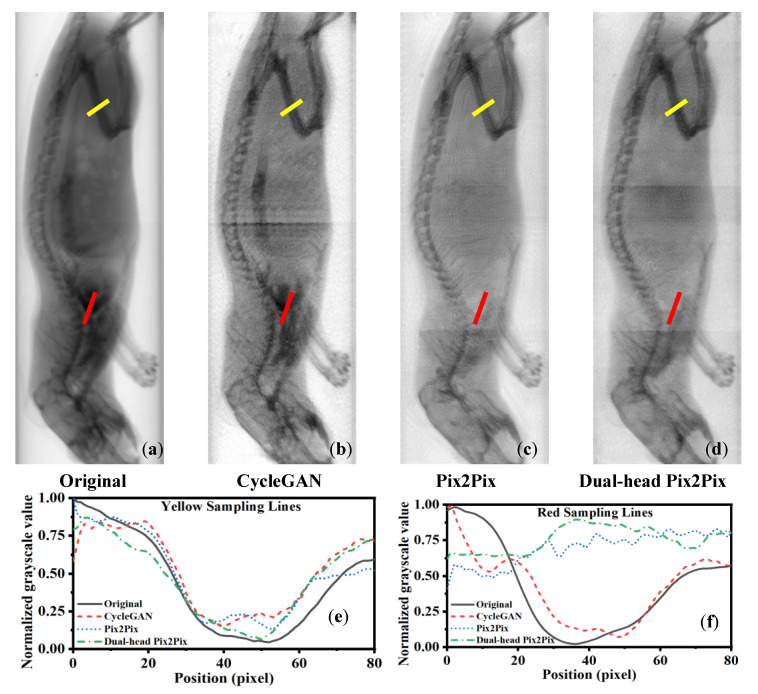
Line profile analysis of ROIs in bone decomposition results obtained with different algorithms. (**a**) Original input; (**b**) result from CycleGAN; (**c**) result from Pix2Pix; and (**d**) result from Dual-head Pix2Pix. The yellow and red lines in (**a**–**d**) indicate the selected ROIs, where the yellow line corresponds to the bone region and the red line corresponds to the iodine region. (**e**) Line profile of the yellow ROI; (**f**) line profile of the red ROI.

Figure 6 shows the region-specific line profile analysis for iodine decomposition using different algorithms. The yellow and red sampling lines indicate bone-rich and iodine-rich regions, respectively. In the yellow line profiles (e), a higher similarity to the input denotes better bone preservation; Dual-head Pix2Pix achieves the closest match. In the red line profiles (f), greater deviation signifies better iodine suppression. Dual-head Pix2Pix again shows the largest difference, indicating the most effective removal of iodine artifacts.

### 4.2. Traditional EID Projection Image Cross-Domain Testing

**Figure 7:**
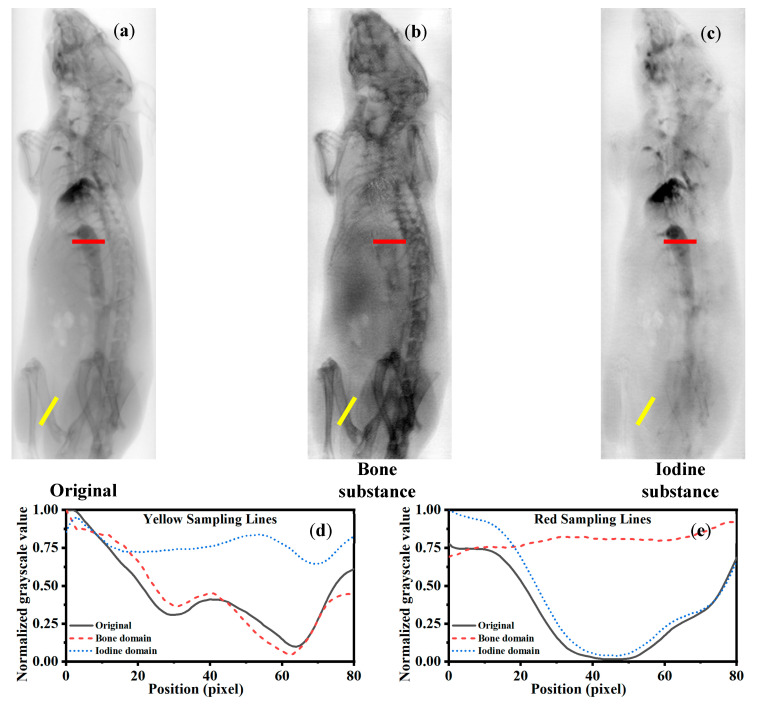
Performance evaluation of the Dual-head Pix2Pix model on projection images from conventional EIDs. (**a**) Original projection image from EID, (**b**) line profile along yellow line (bone region), (**c**) line profile along red line (iodine region). The yellow and red lines in (**a**–**c**) indicate the selected ROIs, where the yellow line corresponds to the bone region and the red line corresponds to the iodine region. (**d**) Line profile of the yellow ROI; (**e**) line profile of the red ROI.

To evaluate the generalizability of the proposed method across different acquisition devices and imaging conditions, cross-domain testing was conducted using projection images acquired with traditional EID. These EID images exhibit different signal characteristics and noise distributions compared to the images used during model training. The cross-domain evaluation aims to assess the model’s robustness and applicability on unseen data distributions. By comparing decomposition results between the training-domain and the EID-domain projections, we analyze the stability and potential practical value of the method in diverse imaging scenarios. The acquired mouse angiography images (Figure 7a) were preprocessed and cropped before undergoing material decomposition using the Dual-head Pix2Pix model. As shown in Figure 7b,c, the decomposition results are based on data acquired by a conventional energy-integrating detector. Unlike dual-energy systems, these detectors cannot directly produce material decomposition images through energy thresholding, which are considered the “gold standard.” Therefore, the quality of the generated images was evaluated by the retention of the target material in its corresponding decomposition image and the suppression of irrelevant materials.

In Figure 7b, the yellow sampling line marks a bone-rich region. Ideally, the pixel intensities in the bone decomposition image should closely match those in the input projection along this line, while the iodine decomposition image should exhibit minimal bone-related signals. The results show that the bone decomposition image from the Dual-head Pix2Pix aligns well with the input intensities, whereas the iodine decomposition image maintains a uniform intensity, indicating effective bone suppression.

In Figure 7c, the red sampling line indicates an iodine-rich region. In this case, the iodine decomposition image preserves the input signal along the line, while the bone decomposition image displays low and uniform intensities, confirming the successful separation of iodine and bone components.

### 4.3. Ablation Study on Loss Functions in the Dual-Head Pix2Pix Network

To evaluate the effectiveness of the proposed mutual exclusivity loss $L_{3}^{*}$, ablation experiments were conducted using different combinations of loss functions in the Dual-head Pix2Pix network. The performance of iodine and bone material decomposition was evaluated based on four quantitative metrics: MAE, MS-SSIM, Pearson-R, and PSNR.

From Table 3 and Table 4, it can be observed that incorporating the mutual exclusivity loss $L_{3}^{*}$ leads to consistent improvements across all evaluation metrics for both iodine and bone decomposition. While the improvements in MAE and PSNR are relatively modest, the increases in MS-SSIM and Pearson-R suggest more consistent and structurally accurate outputs, thereby confirming the effectiveness of the proposed constraint.

**Figure 8:**
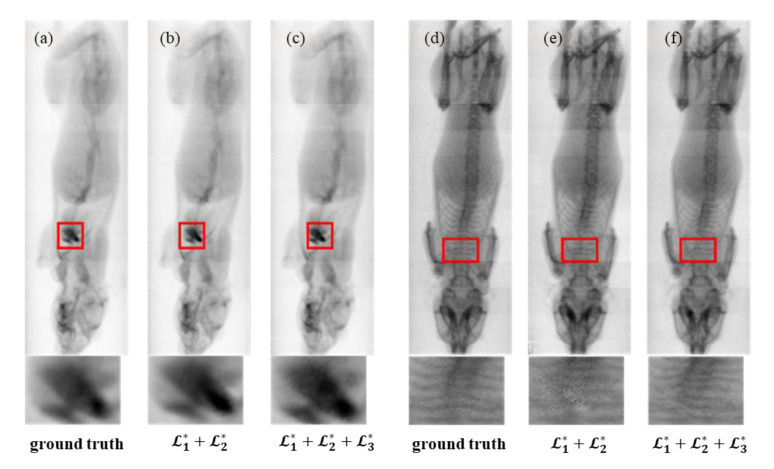
Ablation study results, (**a**) and (**d**) show reference images for iodine and bone decomposition, respectively, (**b**,**e**) display the predicted results without the mutual exclusivity loss $L_{3}^{*}$, while (**c**,**f**) present the predictions with $L_{3}^{*}$ included. Red boxes highlight regions with noticeable differences, particularly in areas where bone and iodine signals overlap.

As shown in Figure 8, models trained with the mutual exclusivity loss $L_{3}^{*}$ produce outputs that are more consistent with the Ground Truth, particularly in anatomically complex regions such as the heart, where iodine and bone signals intersect. The generated images also exhibit smoother textures and improved anatomical separation.

## 5. Discussion

In this study, we proposed the Dual-head Pix2Pix network, which employs a dual-decoder architecture to effectively achieve the material decomposition of iodine and bone from single-energy X-ray projection images. Compared to traditional single-decoder models, the dual-decoder design enables the independent learning of distinct material channels, thereby mitigating interference caused by signal overlaps between materials. Although the introduced mutual exclusivity loss term $L_{3}^{*}$ yielded moderate improvements in global quantitative metrics, it played a critical role in enhancing fine detail preservation and boundary clarity, particularly in anatomically complex regions where iodine and bone signals overlap. Both ablation studies and qualitative evaluations corroborate the effectiveness of this design (Table 3 and Table 4 and Figure 8).

Quantitative assessments across multiple metrics—including MAE, MS-SSIM, Pearson correlation coefficient, and PSNR—demonstrate that the Dual-head Pix2Pix model consistently outperforms CycleGAN and the standard Pix2Pix model, achieving a superior image reconstruction accuracy and structural similarity (Table 1 and Table 2 and Figure 4, Figure 5 and Figure 6). Furthermore, cross-domain evaluations (Figure 7) validate the model’s generalization capability on projection images acquired by conventional energy-integrating detectors, which lack true dual-energy references. Despite this limitation, the model successfully preserved target material signals while suppressing irrelevant content, underscoring its potential for practical application in clinical and industrial settings with low-cost detectors.

Furthermore, as observed in Figure 4, Figure 5 and Figure 6 and Figure 8, both the Ground Truth and the network-generated iodine–bone separation results derived from PCD-CT projection data exhibit horizontal striped artifacts. These artifacts likely arise from multiple factors, including photon starvation, noise amplification, and beam-hardening effects. Material decomposition algorithms require the simultaneous use of low- and high-energy data to resolve iodine and calcium densities. The extremely high noise present in the low-energy data becomes significantly amplified during the decomposition process. When the algorithm attempts to interpret this unstructured noise, it erroneously attributes these fluctuations to slight variations in iodine or calcium signals, resulting in distinct, alternating striped artifacts perpendicular to the X-ray propagation direction. Notably, as shown in Figure 7, when the proposed method is applied to material decomposition using projection images acquired with EID-CT, the horizontal striped artifacts are eliminated. This suggests that our approach can effectively suppress such striped artifacts and improve image quality in material decomposition when processing EID-CT projection data. While our proposed network demonstrated accurate material decomposition on the tested datasets, several limitations should be noted. First, the network has been trained and evaluated on projection images from phantoms and external EID system data, showing no signs of overfitting. However, its performance on larger animals or clinical subjects remains untested. Future work will focus on validating the method in more complex, heterogeneous objects to assess its applicability beyond the current datasets.

In this study, we intentionally performed material decomposition in the projection domain rather than directly on reconstructed CT slices. Our aim was to investigate whether projection-domain learning using data from a conventional EID system can approximate the material separation performance of PCD systems. Compared with image-domain decomposition, projection-domain approaches preserve richer structural and spectral information, enable the incorporation of physical constraints at an early stage, and help reduce error accumulation, suppress beam-hardening artifacts, and compensate for detector- and acquisition-related distortions.

Although only projection-domain decomposition was presented in this work, this represents the first stage of our research. In future studies, we plan to extend this framework to image reconstruction based on decomposed projections, thereby achieving slice-level material decomposition (e.g., reconstructed BoneProj and IodineProj slices). This workflow is consistent with the principles of PCD-CT systems and will be the focus of our subsequent research.

Nonetheless, several limitations persist. The model training relies on paired dual-energy reference data, which may restrict applicability in scenarios where such annotations are unavailable or limited. Residual artifacts remain in certain challenging regions, suggesting that the further refinement of loss functions and network architecture is necessary. Moreover, computational efficiency and inference speed require improvement to meet the demands of real-time or large-scale deployment.

Future work will focus on exploring weakly supervised and unsupervised learning strategies to reduce dependency on paired training data, integrating domain adaptations and transfer learning techniques to enhance robustness across varying acquisition conditions and devices, and optimizing network design to improve computational performance and scalability.

In summary, the Dual-head Pix2Pix framework offers an effective AI-driven solution for single-energy material decomposition, improving accuracy and image quality while bridging the functional gap between low-cost conventional detectors and advanced photon-counting systems. This work lays a solid foundation for expanding material-specific imaging capabilities in both clinical and industrial applications without necessitating hardware upgrades.

## 6. Conclusions

In this work, we proposed the Dual-head Pix2Pix network for iodine and bone material decomposition from X-ray projection images. By modifying the generator architecture and introducing a mutual exclusivity loss, the network achieves superior material separation compared to traditional models. Experimental results demonstrate improvements in both the accuracy and visual quality of the decomposition images. Moreover, the model generalizes data acquired from conventional detectors well. Future work will focus on extending this approach to CT-reconstructed images to enable a more accurate 3D material decomposition.

## Figures and Tables

**Table 1:** Results of quantitative assessment of iodine substance decomposition using different methods.

Models	MAE	MS-SSIM	Pearson-R	PSNR
Original	51.07 ± 11.45	0.80 ± 0.04	0.91± 0.03	13.01 ± 1.78
Cycle GAN	10.39 ± 4.05	0.86 ± 0.05	0.96 ± 0.02	26.40 ± 2.65
pix2pix	5.92 ± 3.45	0.90 ± 0.04	0.98 ± 0.01	31.19 ± 2.62
Dual-head Pix2Pix	5.30 ± 1.81 *	0.91 ± 0.03 *	0.99 ± 0.01	32.06 ± 2.62 *

* Paired *t*-tests showed that Dual-head Pix2Pix significantly outperformed all other methods across all metrics (*p* < 0.0001).

**Table 2:** Results of quantitative assessment of bone substance decomposition using different methods.

Model	MAE	MS-SSIM	Pearson-R	PSNR
Input	22.77 ± 9.99	0.78 ± 0.07	0.94 ± 0.04	19.72 ± 3.05
CycleGAN	21.19 ± 5.84	0.70 ± 0.06	0.92 ± 0.04	19.92 ± 1.97
pix2pix	10.18 ± 2.88	0.83 ± 0.03	0.98 ± 0.01	26.20 ± 1.90
Dual-head Pix2Pix	9.55 ± 2.49 *	0.84 ± 0.03 *	0.98 ± 0.01	26.74 ± 1.97 *

* Paired *t*-tests showed that Dual-head Pix2Pix significantly outperformed all other methods across all metrics (*p* < 0.0001).

**Table 3:** Quantitative evaluation of iodine decomposition with different loss function combinations.

Loss Function	MAE	MS-SSIM	Pearson-R	PSNR
$L_{1}^{*}+L_{2}^{*}$	5.30 ± 2.04	0.90 ± 0.04	0.98 ± 0.01	31.95 ± 2.49
$L_{1}^{*}+L_{2}^{*}+L_{3}^{*}$	5.30 ± 1.81	0.91 ± 0.03	0.99 ± 0.01	32.06 ± 2.62

**Table 4:** Quantitative evaluation of bone decomposition with different loss function combinations.

Loss Function	MAE	MS-SSIM	Pearson-R	PSNR
$L_{1}^{*}+L_{2}^{*}$	9.68 ± 2.64	0.83 ± 0.03	0.98 ± 0.01	26.63 ± 2.07
$L_{1}^{*}+L_{2}^{*}+L_{3}^{*}$	9.55 ± 2.49	0.84 ± 0.03	0.98 ± 0.01	26.74 ± 1.97

## Data Availability

The data that supports the findings of this study are available from the corresponding author, Shouping Zhu, upon reasonable request.

## Abbreviations

The following abbreviations are used in this manuscript:

PCD	Photon-counting detector
MAE	Mean absolute error
CT	Computed tomography
EIDs	Energy-integrating detectors
DECT	Dual-energy CT
PCDs	Photon-counting detectors
MS-SSIM	Multi-scale structural similarity index
Pearson-R	Pearson correlation coefficient
PSNR	Peak signal-to-noise ratio
ROI	Regions of interest
